# Effect of Diagnostic Test Type on Detection of *Salmonella* Infections — Foodborne Diseases Active Surveillance Network, 2004–2024

**DOI:** 10.15585/mmwr.mm7529a2

**Published:** 2026-07-30

**Authors:** Reese Tierney, Robin Pendley Louis, David J. Boxrud, Kennedy Houck Lamas, Kristina M. Angelo, Daniel L. Weller

**Affiliations:** 1 Division of Foodborne, Waterborne and Environmental Diseases, National Center for Emerging and Zoonotic Infectious Diseases, CDC.

**Summary**

**What is already known about this topic?**

Despite increasing use of culture-independent diagnostic tests (CIDTs), the impact of these tests on *Salmonella* epidemiology has not been evaluated.

**What is added by this report?**

Overall *Salmonella* incidence in the Foodborne Diseases Active Surveillance Network catchment area remained stable during 2004–2024, but diagnostic patterns shifted substantially. The incidence of culture-diagnosed infections declined as CIDT-diagnosed infection incidence increased. Mild and sporadic infections were more likely than were severe and outbreak-associated infections to be diagnosed by CIDTs.

**What are the implications for public health practice?**

Increasing CIDT use allows for improved detection of mild and sporadic infections but might obscure decreases in disease incidence from successful prevention efforts. Surveillance systems and prevention strategies should account for evolving diagnostic practices while maintaining efforts to recover isolates for public health action.

## Abstract

*Salmonella* species bacterial infections are a leading cause of enteric illness and can be diagnosed using bacterial culture or culture-independent diagnostic tests (CIDTs). All culture-diagnosed cases are considered culture confirmed. After a positive CIDT, infection can be culture confirmed if reflex culture is attempted and yields an isolate. A culture-unconfirmed infection is a CIDT-diagnosed infection for which reflex culture was not attempted or failed to yield an isolate. Foodborne Diseases Active Surveillance Network data from 2006 through 2023 were analyzed to compare the epidemiologic and clinical characteristics of *Salmonella* infections after stratifying by diagnostic method (culture versus CIDT) and culture confirmation status (culture confirmed versus unconfirmed). To improve the precision and stability of temporal trend estimates, incidence trends during 2004–2024, as opposed to 2006–2023, were evaluated overall, by diagnostic method, and by confirmation status using regression. Although overall incidence remained stable from 2004 through 2024, the incidence of culture-diagnosed and -confirmed infections declined, and the incidence of CIDT-diagnosed and unconfirmed infections increased. Culture- and CIDT-diagnosed *Salmonella* infections were demographically similar but exhibited distinct clinical patterns, with CIDT diagnosis associated with milder and more sporadic illnesses, which suggests that these infections were historically underdiagnosed by culture-based diagnostics. The success of prevention efforts that reduce more severe, traditionally culture-detected *Salmonella* infections might be offset by the expanded detection of milder infections through CIDTs. The increases in detection of milder infections through CIDTs may offset the results of successes in prevention efforts in reduction in severe Salmonella infections. Understanding how diagnostic methods influence observed surveillance trends is therefore critical for evaluating and guiding future prevention efforts.

## Introduction

*Salmonella* species bacterial infections are a leading cause of enteric gastrointestinal disease in the United States and are spread through consumption of or contact with contaminated food or water or contact with infected persons or animals. *Salmonella* infection can be categorized as nontyphoidal *Salmonella* (NTS) or typhoidal *Salmonella* (TS). TS infections include typhoid fever and *Paratyphi* infections. These infections accounted for approximately 1% (1,496 of 107,207) of *Salmonella* infections in the United States during 2004–2024, and approximately 70% of these TS infections were associated with international travel (1,031 of 1,496 during 2004–2024). In contrast, NTS is the second leading cause of domestically acquired bacterial foodborne illness in the United States, causing an estimated 1.3 million illnesses and 12,500 hospitalizations in 2019 (*1*,*2*). One objective of Healthy People 2030 is to reduce domestically acquired NTS infection to fewer than 11.5 infections per 100,000 population by 2030 (*3*). The incidence of domestically acquired NTS infection in the Foodborne Diseases Active Surveillance Network (FoodNet) was 15.2 infections per 100,000 population in 2024, which might suggest that limited progress has been made toward this goal (*3*).

Historically, *Salmonella* infection, whether NTS or TS, has been diagnosed using culture-based methods. Culture-independent diagnostic tests (CIDTs), which detect a pathogen’s genetic material or antigens, have been increasingly used since 2013, are often performed as part of multiplex panels, and can identify enteric infections without requiring pathogen-specific clinical suspicion (*4*). After a CIDT diagnosis, infections can be culture confirmed.* For several enteric pathogens, increasing CIDT use has been associated with higher reported incidence and changes in the characteristics of reported infections (*2*,*5*). Consequently, changes in diagnostic practices can complicate interpretation of illness trends and assessment of progress toward disease reduction goals (*5*,*6*). To assess how evolving diagnostic practices affect *Salmonella* epidemiology, this report describes *Salmonella* infection incidence and characteristics for illnesses reported in the FoodNet catchment area by diagnostic method and confirmation status.

## Methods

### Data Source

During 2004–2023, the FoodNet catchment area included Connecticut, Georgia, Maryland, Minnesota, New Mexico, Oregon, and Tennessee and selected counties in California, Colorado, and New York (i.e., the historic FoodNet catchment area). In 2023, the FoodNet catchment area expanded to include all Colorado counties (i.e., the expanded FoodNet catchment area) (*2*).

FoodNet has conducted active, population-based surveillance for culture-diagnosed infections since 1996 and, since 2012, for CIDT-diagnosed infections caused by eight enteric pathogens, including *Salmonella,* in the FoodNet catchment area^†^ (*2*). After a CIDT diagnosis, infections are considered culture confirmed if reflex culture is attempted and yields an isolate. All culture-diagnosed cases are considered culture confirmed. An unconfirmed infection is an infection for which reflex culture was not attempted or failed to yield an isolate.

For each illness, FoodNet collects demographic, clinical, specimen type, and other data, including whether the illness is outbreak-associated or sporadic, and whether the person reported international travel <30 days before TS infection onset or <7 days before NTS infection onset. To diagnose a *Salmonella* infection, various specimens can be tested, including blood, stool, and urine. Data for all *Salmonella* infections reported to FoodNet during 2004–2024 (NTS and TS) were obtained for use in this report, although slightly different ranges of years were used, depending on the analysis.

### Statistical Methods

All data were analyzed in R (version 4.4.1; R Foundation). Sensitivity analyses excluded persons reporting international travel and used data from the expanded FoodNet catchment area. Because these analyses produced similar results, findings from the historic FoodNet catchment area, including travel-associated cases, are included.

The demographic and clinical characteristics of infections reported during 2006–2014 and 2015–2023 were compared after stratification by diagnostic method and confirmation status. These years were selected to reflect the period immediately before and after Food and Drug Administration (FDA) approval of the first CIDT multiplex panel with a *Salmonella* target in 2013 and to allow for equal time periods for comparison before and after CIDT adoption.^§^ Demographic and clinical characteristics were not available for 2024 at the time of this analysis. For each period, the average annual incidences (number of cases per 100,000 population) were calculated using U.S. Census Bureau intercensal population estimates.^¶^ Incidence rate ratios (IRRs) with 95% CIs were used to compare incidences across periods and stratum. Counterfactual random forest (CFRF),** a method to quantify differences in odds of a case being diagnosed by CIDT (versus culture) for different demographic patient and clinical illness characteristics, was also conducted^††^ (*5*,*7*).

Bayesian splines regression^§§^ was used to model trends in culture-diagnosed and culture-confirmed infections during 2004–2024 and in CIDT-diagnosed and unconfirmed infections during 2011–2024^¶¶^ (*8*,*9*). All available data from 2004–2024 were used to identify long-term trends and improve estimate precision. This activity was reviewed by CDC, deemed not research, and conducted consistent with applicable federal law and CDC policy.***

## Results

During 2004–2024, the overall *Salmonella* infection incidence remained stable. The incidence of culture-diagnosed and culture-confirmed infections decreased, and the incidence of CIDT-diagnosed and unconfirmed infections increased (Figure).^†††^ The number of infections diagnosed by CIDT exceeded those diagnosed by culture in 2021.^§§§^

**FIGURE:**
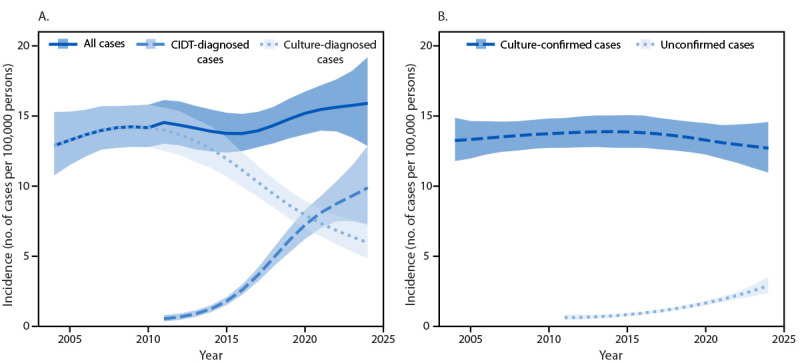
Trend in the estimated incidence* of all *Salmonella* infections, infections diagnosed by culture and culture-independent diagnostic tests (A), and culture-confirmed and unconfirmed *Salmonella* infections (B) — Foodborne Diseases Active Surveillance Network, 2004–2024 **Abbreviation**: CIDT = culture-independent diagnostic test. * All culture-diagnosed infections were considered culture confirmed; CIDT-diagnosed infections were considered confirmed if a reflex culture after CIDT diagnosis was performed and a *Salmonella* isolate was obtained. An infection was considered unconfirmed if reflex culture was not attempted or failed to yield an isolate. All available data from 2004–2024, as opposed to 2006–2023, were used to identify long-term trends and improve estimate precision.

During 2006–2014 and 2015–2023, a total of 66,993 infections (incidence = 15.8 infections per 100,000 population) and 73,254 infections (incidence = 16.12) were reported, respectively (Table 1) (Supplementary Table 1). Overall, 66,281 infections (incidence = 15.70) and 44,942 infections (incidence = 9.89) during 2006–2014 and 2015–2023, respectively, were culture diagnosed (IRR = 0.63), whereas 712 infections (incidence = 0.17) and 28,312 infections (incidence = 6.23) were CIDT diagnosed (IRR = 36.65) (Table 1) (Supplementary Table 2). A total of 312 infections (incidence = 0.07) during 2006–2014 and 8,448 infections (incidence = 1.86) during 2015–2023 were not culture confirmed (IRR = 26.57) (Supplementary Table 3). Although the incidence of culture-confirmed infections decreased from 2006–2014 (incidence = 15.70) to 2015–2023 (incidence = 14.30), the magnitude of the decrease was small (IRR = 0.91).

**TABLE 1:** Demographic characteristics of patients with *Salmonella* species infections diagnosed by culture-based tests or culture-independent diagnostic tests — Foodborne Diseases Active Surveillance Network catchment area, 2006–2014 and 2015–2023

Characteristic	2006–2014				2015–2023				IRR, 2015−2023 vs. 2006−2014 (95% CI)	
	Cx		CIDT^†^		Cx		CIDT^†^		Cx	CIDT^†^
	No. (%)	Incidence*	No. (%)	Incidence*	No. (%)	Incidence*	No. (%)	Incidence*		
**Total, no.**	**66,281**	**15.70**	**712**	**0.17**	**44,942**	**9.89**	**28,312**	**6.23**	**0.63 (0.62–0.64)**	**36.65 (34.02–39.48)**
**Age group, yrs**										
<1	6,535 (10)	13.54	121 (17)	0.25	3,301 (7)	7.20	2,769 (10)	6.04	0.53 (0.51–0.56)	24.16 (20.14–28.98)
1–4	10,742 (16)	5.51	178 (25)	0.09	5,443 (12)	2.89	4,156 (15)	2.20	0.21 (0.21–0.22)	24.44 (21.04–28.4)
5–17	10,436 (16)	1.59	124 (17)	0.02	5,386 (12)	0.81	3,538 (12)	0.53	0.51 (0.49–0.53)	26.50 (22.16–31.7)
18–59	27,470 (41)	1.24	214 (30)	0.01	19,421 (43)	0.85	12,141 (43)	0.53	0.69 (0.67–0.70)	53.00 (46.3–60.67)
≥60	11,054 (17)	1.59	75 (11)	0.01	11,389 (25)	1.25	5,708 (20)	0.63	0.79 (0.77–0.81)	63.00 (50.17–79.12)
Not reported	44 (<0.1)	—^§^	1 (<0.1)	—	2 (<0.1)	—	0 (—)	—	—	—
**Ethnicity**										
Hispanic or Latino	6,246 (9)	13.31	52 (7)	0.11	4,697 (10)	8.10	3,783 (13)	6.53	0.61 (0.59–0.63)	59.36 (45.15–78.05)
Not Hispanic or Latino	45,873 (69)	12.18	529 (74)	0.14	34,916 (78)	8.81	21,672 (77)	5.47	1.38 (1.36–1.40)	39.07 (35.84–42.59)
Not reported	14,161 (21)	—	131 (18)	—	5,329 (12)	—	2,857 (10)	—	—	—
**Race**										
American Indian or Alaska Native	648 (1)	12.87	1 (0.1)	0.02	450 (1)	7.65	209 (1)	3.55	0.59 (0.53–0.67)	177.50 (24.89–1266.05)
Asian	3,002 (5)	14.04	38 (5)	0.18	1,957 (4)	7.09	1,376 (5)	4.98	0.50 (0.48–0.53)	27.67 (20.04–38.19)
Black or African American	9,116 (14)	13.53	55 (8)	0.08	6,175 (14)	8.13	3,487 (12)	4.59	0.60 (0.58–0.62)	57.38 (43.96–74.89)
Native Hawaiian or Pacific Islander	83 (<1)	20.55	1 (<1)	0.25	94 (<1)	10.90	68 (<1)	7.89	0.53 (0.39–0.71)	31.56 (4.38–227.3)
White	41,932 (63)	13.11	477 (67)	0.15	29,565 (66)	8.93	19,005 (67)	5.74	0.68 (0.67–0.69)	38.27 (34.94–41.91)
Multiple races	648 (1)	6.83	5 (1)	0.05	481 (1)	3.73	446 (2)	3.46	0.55 (0.49–0.61)	69.20 (28.66–167.07)
Other race	1,443 (2)	—	12 (1.7)	—	1,827 (4)	—	1,537 (5)	—	—	—
Not reported	9,409 (14)	—	123 (17)	—	4,393 (10)	—	2,184 (8)	—	—	—
**Sex**										
Female	34,572 (52)	16.02	352 (49)	0.16	24,244 (54)	10.51	14,804 (52)	6.42	0.66 (0.65–0.67)	40.12 (36.10–44.6)
Male	31,603 (48)	15.21	358 (50)	0.17	20,570 (46)	9.19	13,435 (47)	6.00	0.60 (0.59–0.61)	35.29 (31.78–39.20)
Not reported	106 (<1)	—	2 (<1)	—	128 (<1)	—	73 (<1)	—	—	—

**Abbreviations:** CIDT = culture-independent diagnostic test; Cx = culture; IRR = incidence rate ratio.

* Incidence per 100,000 population was calculated by dividing the number of infections during a given year by the U.S. Census Bureau census or intercensal population estimates for that year using the calculate_average_annual_incidence function in the epi-toolbox-r repository.

^†^ Includes cases diagnosed by CIDT only (CIDT+ [i.e., no reflex Cx]) or by CIDTs with either a positive reflex Cx (CIDT+/Cx+) or negative reflex Cx (CIDT+/Cx–).

^§^ Dashes indicate that data were not reported or were otherwise missing from the Foodborne Diseases Active Surveillance Network database. U.S. Census Bureau data used to describe catchment area characteristics do not include a comparable “not reported” category for any of the characteristics of interest.

Infections identified using blood specimens (odds ratio [OR] = 0.02) or urine specimens (OR = 0.03), rather than stool specimens, had lower odds of being diagnosed by CIDT than by culture (Table 2). Other indicators of severe infection (i.e., hospitalization or having bloody diarrhea) were also associated with lower odds of CIDT diagnosis, although effect sizes were closer to 1.0. Odds of CIDT diagnosis were lower for outbreak-associated versus sporadic infections (OR = 0.20). Despite statistically significant associations, ORs for demographic characteristics were generally close to 1.0, suggesting minimal demographic differences between CIDT-diagnosed and culture-diagnosed infections.

**TABLE 2:** Odds that a *Salmonella* species infection was diagnosed by a culture-based test or a culture-independent diagnostic test — Foodborne Diseases Active Surveillance Network, 2006–2023

Characteristic	OR	95% CI	p-value
**Clinical characteristics**			
Hospitalized*			
No	Ref	—	—
Yes	0.78	(0.75−0.82)	<0.001
Not reported	1.42	(1.30−1.56)	<0.001
Illness type			
Sporadic	Ref	—	—
Outbreak associated^†^	0.20	(0.17−0.24)	<0.001
Patient status			
Alive	Ref	—	—
Dead^§^	1.25	(0.95−1.64)	0.11
Not reported	2.20	(2.03−2.38)	<0.001
Specimen type			
Stool or rectal swab specimen	Ref	—	—
Blood	0.02	(0.01−0.03)	<0.001
Urine	0.03	(0.02−0.04)	<0.001
Other	0.89	(0.76−1.04)	0.14
Not reported	1.09	(0.84−1.43)	0.52
**Signs and symptoms^¶^**			
Bloody diarrhea			
No	Ref	—	—
Yes	0.84	(0.80−0.89)	<0.001
Not reported	0.71	(0.68−0.75)	<0.001
Any diarrhea			
No	Ref	—	—
Yes	0.64	(0.59−0.69)	<0.001
Not reported	0.92	(0.85−0.99)	0.03
Fever			
No	Ref	—	—
Yes	0.69	(0.66−0.73)	<0.001
Not reported	0.80	(0.76−0.84)	<0.001
**Demographic characteristics**			
Age group, yrs			
<1	Ref	—	—
1–4	0.89	(0.82−0.96)	0.002
5–17	0.70	(0.64−0.76)	<0.001
18–59	0.62	(0.58−0.66)	<0.001
≥60	0.68	(0.63−0.74)	<0.001
Ethnicity			
Hispanic or Latino	1.09	(1.02−1.17)	0.01
Not Hispanic	Ref	—	—
Not reported	1.18	(1.12−1.25)	<0.001
Race			
American Indian or Alaska Native	0.90	(0.71−1.14)	0.38
Asian	1.38	(1.26−1.51)	<0.001
Black or African American	1.08	(1.01−1.15)	0.02
White	Ref	—	—
Multiple races	1.36	(1.14−1.62)	0.001
Other race	1.15	(1.03−1.28)	0.01
Not reported	1.39	(1.31−1.48)	<0.001
Sex			
Female	Ref	—	—
Male	0.95	(0.91−1.00)	0.03
Urbanicity**			
Large metropolitan	Ref	—	—
Large metropolitan fringe (i.e., suburban)	1.36	(1.28−1.44)	<0.001
Medium metropolitan	1.01	(0.93−1.08)	0.89
Small metropolitan	1.02	(0.93−1.10)	0.72
Micropolitan	1.12	(1.04−1.21)	0.003
Noncore (i.e., rural)	1.24	(1.14−1.36)	<0.001
**Season**			
Summer	Ref	—	—
Fall	1.03	(0.98−1.08)	0.30
Spring	1.02	(0.96−1.09)	0.45
Winter	1.05	(0.99−1.12)	0.11
**Site^††^**			
California	1.66	(1.55−1.78)	<0.001
Colorado	1.08	(0.98−1.18)	0.13
Connecticut	0.65	(0.58−0.72)	<0.001
Georgia	1.31	(1.25−1.37)	<0.001
Maryland	1.01	(0.95−1.08)	0.75
Minnesota	0.87	(0.81−0.93)	<0.001
New Mexico	0.62	(0.55−0.70)	<0.001
New York	0.82	(0.74−0.90)	<0.001
Oregon	1.00	(0.91−1.10)	0.98
Tennessee	1.75	(1.66−1.85)	<0.001
**Travel status**			
Domestically acquired	Ref	—	—
Travel associated^§§^	1.15	(1.07−1.24)	<0.001
Not reported	1.26	(1.20−1.32)	<0.001
**Years**			
2006–2014 (before CIDTs and laboratory-developed CIDTs)	Ref	—	—
2015–2017 (early FDA-approved CIDT)	8.43	(7.72−9.20)	<0.001
2018–2019 (prepandemic)	13.27	(12.17−14.48)	<0.001
2020–2021 (COVID-19 pandemic onset)	17.29	(15.85−18.87)	<0.001
2022–2023 (high CIDT adoption)	22.85	(21.02−24.84)	<0.001

**Abbreviations:** CIDT = culture-independent diagnostic test; FDA = Food and Drug Administration; OR = odds ratio; Ref = referent group.

* Defined as admission to a hospital inpatient unit or an observation stay of >24 hours ≤7 days before or after specimen collection or determined to be related to the infection if beyond this time frame.

^†^ Defined as two or more cases of similar illness associated with a common exposure; some sites also stipulate that illnesses be from one or more households.

^§^ Attributed to infection when death occurred during hospitalization or ≤7 days after specimen collection from nonhospitalized patients.

^¶^ Data on signs and symptoms were collected beginning in 2011.

** Urbanicity was determined using the National Center for Health Statistics rural-urban classification scheme for the patient’s county of residence.

^††^ Site variables were dummy variables with values of yes (the infection occurred in a resident of the given state) or no (the infection did not occur in a resident of the given state).

^§§^ If the patient did not report international travel or had an unknown travel history, the illness was considered to have been domestically acquired. History of international travel was defined as travel ≤7 days before onset of nontyphoidal infections and ≤30 days before onset of typhoidal infections.

## Discussion

Overall *Salmonella* infection incidence remained stable during 2006–2023; however, substantial shifts in clinical testing practices occurred with increasing use of CIDTs and decreasing reliance on culture-based diagnosis over time. Geographic differences in odds of CIDT diagnosis likely reflect variation in CIDT adoption by laboratories serving different communities (*5**,**8*).

Although demographic characteristics of culture- and CIDT-diagnosed infections were similar, they differed by severity and signs and symptoms. Mild and sporadic illnesses were more likely to be CIDT diagnosed, whereas severe (e.g., bloodstream) and outbreak-associated illnesses were more often culture diagnosed. This pattern might reflect the common clinical practice of ordering blood cultures for patients with signs and symptoms of sepsis. Overall, these findings indicate that CIDTs might have altered the spectrum of *Salmonella* illnesses detected without fundamentally changing the demographic profile of reported *Salmonella* cases. As a result, CIDTs might detect mild and sporadic illnesses (or infections) that previously would have remained undiagnosed (*4*), increases in CIDT-diagnosed infections might mask reductions in illnesses resulting from successful prevention and control efforts. Diagnostic methods should be accounted for in data analyses, for example, through stratification in epidemiologic investigations and inclusion as a covariate in models.

The slower decline in culture-confirmed (versus culture-diagnosed) infections might suggest reflex culture rates are not decreasing at the same rate CIDT use is increasing, although other factors could contribute to this pattern. For example, *Salmonella* is relatively easy to culture, which might facilitate recovery of isolates after CIDT diagnosis. Maintaining high reflex culture rates after CIDT-diagnosed *Salmonella* infections remains important, because isolates are required for serotyping, whole-genome sequencing (WGS), and antimicrobial susceptibility testing. WGS, in turn, is critical for outbreak detection, source attribution, and other public health surveillance activities.

### Limitations

The findings in this report are subject to at least three limitations. First, because FoodNet surveillance includes laboratory-diagnosed infections, changes in care-seeking behaviors or testing practices over time can influence observed trends in infection incidence. Second, infections detected by CIDT were classified as CIDT-positive regardless of reflex culture results, potentially misclassifying infections because of false-positive CIDT results, although false-positive CIDT results are uncommon for *Salmonella* (*10*). Finally, because of the number of unserotyped infections, NTS and TS were not considered separately; instead, a sensitivity analysis excluding travel-associated infections was performed because 70% (1,031 of 1,496 cases) of TS infections compared with 8% (7,935 of 105,711) of NTS infections were travel associated during 2004–2024. Results from the sensitivity analysis were comparable to the primary analysis, suggesting inclusion or exclusion of travel-associated cases, including most TS cases, did not alter the conclusions.

### Implications for Public Health Practice

As CIDT use increases, trends in reported *Salmonella* infection incidence should be interpreted in the context of changing diagnostic practices, particularly when comparing trends across time or populations. The increasing use of CIDTs allows for improved detection of mild and sporadic infections, which helps to optimize clinical detection and treatment. However, increasing detection of milder illnesses through CIDTs might obscure declines in more severe infections resulting from successful *Salmonella* infection prevention and control efforts. Performing reflex cultures with CIDT would improve availability of isolates for serotyping, WGS, and antimicrobial susceptibility testing. Continued surveillance, coupled with sustained efforts to recover isolates from CIDT-positive specimens, is important for maintaining effective foodborne disease prevention and response activities. Understanding how evolving diagnostic practices influence observed epidemiologic patterns can improve assessment of progress toward national *Salmonella* reduction goals and help guide prevention strategies.

## Footnotes

* Reflex culture refers to the practice of attempting to isolate a pathogen through culture after a positive CIDT result. Reflex culture practices vary by laboratory, state, and pathogen. Although positive CIDTs do not require a culture for a diagnosis, reflex culture might be attempted after a positive result. Infections diagnosed by CIDT were considered CIDT diagnosed, regardless of reflex culture results. Culture-diagnosed and reflex-culture–positive CIDT-diagnosed infections were considered culture confirmed.

† FoodNet is a collaboration among CDC, 10 state health departments, the U.S. Department of Agriculture, and FDA.

§ Individual laboratories developed and began using CIDTs with a *Salmonella* target in 2012; these laboratory-developed tests were not FDA approved. The first FDA-approved CIDT with a *Salmonella* target was released in 2013. The number and adoption of FDA-approved CIDTs increased from 2013 onwards. Rapid use began in 2015.

¶ Incidence per 100,000 population was calculated by dividing the number of infections during a given year by the U.S. Census Bureau census or intercensal population estimates for that year using the calculate_average_annual_incidence function in the epi-toolbox-r repository. Unknown responses were included in proportion denominators. Missing values were treated as “not reported” for all analyses. Incidence rate ratios were calculated by dividing, for a given stratum, the incidence rate for 2015–2023 by the incidence rate for 2006–2014.

** CFRF builds an ensemble of decision trees to model the relationship between exposures and outcomes. For each observation, it predicts outcomes under both observed and counterfactual exposure scenarios. These predicted probabilities are then used to estimate ORs by contrasting outcome probabilities across exposure conditions.

†† Periods were categorized as 2006–2014 (before CIDTs or when laboratory-developed CIDTs were available); 2015–2017 (early FDA-approved CIDTs); 2018–2019 (prepandemic); 2020–2021 (COVID-19 pandemic onset); and 2022–2023 (high CIDT adoption). This categorization captured increasing CIDT use and COVID-19–related reporting disruptions that might have differentially affected health care seeking and reporting across populations and jurisdictions.

§§ In contrast with frequentist statistics, which involve significance testing, in a Bayesian model, true significance testing is not performed, and differences are determined to be substantial or not based on the magnitude of effect. Although CIDT-diagnosed infections were not reported until 2012, data for 2011 were included as baseline years (i.e., structural zeros) because CIDTs were not yet in use during that period.

¶¶ Log incidence rate was modeled as the sum of group effects (i.e., diagnostic method or culture confirmation status), group-specific trends, and state-specific trends. Population was included as an offset. The separate spline terms (basis dimension = 6) allowed trends to vary by group effect and state. Weakly informative priors were specified: exponential (10) on spline SDs, normal (−9, 0.25) on the intercept, normal (0, 0.5) on coefficients, gamma (10, 2) on the shape parameter, and normal (−2, 1) on the zero-inflation parameter.

*** 45 C.F.R. part 46, 21 C.F.R. part 56; 42 U.S.C. Sect. 241(d); 5 U.S.C. Sect. 552a; 44 U.S.C. Sect. 3501 et seq.

††† In 2004, 2014, and 2024, the estimated incidences per 100,000 population of all *Salmonella* infections were 13.25 (95% credible interval [CrI] = 11.79–14.87), 14.63 (95% CrI = 13.50–15.81), and 15.64 (95% CrI = 13.70–17.67), respectively. In 2004, all *Salmonella* infections were diagnosed using culture-based tests and, thus, were all culture confirmed. In 2014, the estimated incidences per 100,000 population of confirmed and unconfirmed *Salmonella* infections were 13.89 (95% CrI = 12.74–15.04) and 0.75 (95% CrI = 0.62–0.87), respectively. In 2024, the estimated incidences per 100,000 population of confirmed and unconfirmed *Salmonella* infection were 12.70 (95% CrI = 10.97–14.57) and 2.91 (95% CrI = 2.36–3.53), respectively.

§§§ In 2021, the estimated incidences per 100,000 population of culture and CIDT-diagnosed *Salmonella* infections were 7.34 (95% CrI = 6.43–8.42) and 8.10 (95% CrI = 7.02–9.29), respectively.
